# Mr. Christie's Observations on Gun-Shot Wounds

**Published:** 1800-01

**Authors:** 


### *Mr. Chriſtie's Obſervations on Gun-ſhot Wounds.*

[ Continued from our laſt Number, pp. 447. ]

A Private, of the name of Doogen, belonging to the 27th regiment, was ſhot by a muſquet ball entering half way between the right breaſt and axilla, where the ribs ſit pretty cloſe on each each other, and where the bellies of the pectoral muscles are thickest. The wound became consequently deep, and in the entrance of the ball a portion (fortunately the superior one) of a rib was fractured. The man had great anxiety and difficulty of breathing, with a quick and hurried pulse. There was no great hæmorrhage from the wound. I was certain the ball entered the chest; but I thought it likely that the lung had escaped; for, during the few minutes he was with me, he spat up no blood. The wound was dressed as expeditiously, and as securely, as I could. He was sent the same evening to the general hospital, with little hopes of his surviving the accident; but, twelve days afterwards, I heard from a soldier who came from the hospital, that he was still living, and, as he said, doing well and likely to recover, although the ball still remained in the chest. The particulars of this case, so far as inquiries can go, I shall make my business to report at some future period. Is it possible for a ball to remain harmless, or even compatible with life, on so exquisitely sensible a membrane as the pleura?

If the recoveries from wounds penetrating the thorax and abdomen be few, on account of the extensive inflammation which so constantly follows them, fewer still will the recoveries be where the skull is entered. It is remarkable, however, how great an injury the brain will occasionally receive, and long afterwards accommodate itself to the purposes of life, or even be followed by complete recovery. In the general action which occurred on the 2d inst. and in which, from the nature of the ground, (an immense space of high sands in the vicinity of Camperdown) and from the dispersed situation of the troops, it happened that the wounded would be lying singly; and from the impossibility of getting any kind of carriage on the ground, it might be some time until the wounded could be seen or collected. A private in the 27th regiment had been found on the day of action, by a surgeon of another corps, with a wound in the head, lying alone, and, as he said, dying; for, as the brain had been penetrated, he thought it in vain to do any thing more than, for form sake, to apply a bit of lint and wrap a handkerchief round the head, which, of course, would be done in a hurry. Next day, when traversing over extensive sands, to see if any men were still to be found to whom I could afford any assistance, I found this poor man laying by the side of a bents-bush, in a deplorable situation. Not knowing any thing of him, I asked him his regiment and hurt; he answered by putting forth his hand, as if to implore my assistance; he issued a groan which penetrated my heart; he made signs for drink, but this could not be instantly got; a scanty bog here and there was all the water the place afforded for several thousand men, overcome with fatigue, and many lying helplessly wounded. At last a canteen full was got for my patient; distractedly, and with convulsed eagerness, he tried to hold it to his head until it was half empty! I had him conveyed in a blanket by four of his comrades, and this was his state on examination: He was not wholly senseless, for he constantly made signs for water, and would hardly suffer the canteen to be taken away from him; he was not in a coma, or syncope, but apparently in a state between those two; he frequently attempted to articulate, but could not. An officer, who had formerly been his master, came and spoke to him, and to afford him assistance—it filled his eyes with water. On attempting to raise him, he increased his moaning, and pushed his feet downwards, as if to place himself in an easy posture. On washing his face well with cold water, he seemed to relish it; but on attempting to touch his head, there he shewed signs of morbid sensibility; his countenance was pale and ghastly, for *that* side of the face on which the injury was done was paralytic, the corner of the mouth falling downwards; he had, however, the use of all his limbs; his pulse was feeble, depressed, and intermitting. The dry sands had blown the whole night upon his head and cloaths, all bloody; they formed a hard cake around him. On making his body as comfortable as could well be, I removed the handkerchief from his head, and found a musquet shot had carried away a part of one of the parietal bones, nearly about its centre. From the wound, portions of brain were pushed outwards, some in detached pieces, like tea-spoonfuls. As the edges of the wound did not appear very rugged, or at all depressed, and especially as the brain still continued working out, and the poor man making signs in agony to have his head put up, I just applied simple dressing, as gently and securely as I could, putting him up in blankets, with no hope of his recovery, to be conveyed to the general hospital, whither he was taken late in the evening of the 3d instant.

Four days afterwards, i. e. five from the accident, a soldier came from the hospital, who informed me, he then left Shreeves in the same state, or, as he expressed it, raving, and his brains coming out. The confusion which succeeded this, by our retreat from Alkmaer and its neighbourhood, and the consequent movement, or capture, of the general hospital, which was but a temporary one, have entirely prevented me, for the present, from ascertaining the issue of this case. It is more than probable, however, that the inflammation, and consequent suppuration, would shortly put an end to this patient's sufferings; for for ſuch a fatal termination does not appear to be always brought about by mere loſs of brain.

In penetrating wounds of joints, the recovery, if it ever ſucceeds without amputation, is always at leaſt very uncertain and hazardous; when the capſular ligament is divided, and the bone at the ſame time fractured, it is probable immediate amputation, if the patient be not of too robuſt and plethoric a habit, will be always adviſeable. But the point here is nice;—to thoſe therefore, whoſe experience is extenſive, whoſe dread of the ſucceeding tenſion, inflammation, and ſuppuration, is leſs than that of preſent miſchief, I will yield with ſilent ſubmiſſion: at all events, amputation in ſuch wounds can only be adviſeable in thoſe joints below the hip and ſhoulders. When along with fractured bone there alſo happens extenſive laceration of external parts, as happens generally from cannon-ſhot, ſhells, and ſplinters, I ſuppoſe I need hardly mention, no excuſe ſhould warrant the delay of amputation, as this, or a frightful ſloughing, will alone preſerve life.

Belguere, who was chief ſurgeon in the armies, I believe, of the great King of Purſſia, in fulfilling the dictates of his maſter, recommends amputation never to be had recourſe to; and, if I am not miſtaken, ſums up his experience by a ſort of proof that, under his direction, the caſes of recovery of gun-ſhot wounds were more numerous wherein no operation was had recourſe to, than before, when generally practiſed: but German knives are not the firſt in the world. After all, it is plain the caſes of recovery after amputation, in military practice, muſt be comparatively fewer than what follow in civil life, whether in private or public; for the healthy, full, and plethoric habit, ſoldiers and ſailors generally enjoy at the time of receiving their hurts, is found to be inimical to the formation of a kindly ſtump. The ſparer the habit, unleſs carried to exceſs or old age, the eaſier the recovery after amputation. Farther, the injury which the whole frame receives from the ſhock of a cannon-ball, appears to be in ſome caſes ſufficient to fruſtrate our intentions in amputating. The thigh ſhall be carried off half way up, probably not more than a quart of blood will be loſt, but the patient will loſe his ſpirits and ſtrength amazingly. His pulſe, and whole frame, ſhall be tremulous and diſtracted; the bone may be ſhattered high up, a vaſt part of the body of it muſt be taken away, and the patient ſinks under accumulated irritation.

In this place I cannot help remarking, that ſome of the Ruſſian army ſurgeons, either actuated by a miſtaken humanity, or immerſed in a ſavage darkneſs, appear blindly to follow Belguere's plan of treatment; for, in conſequence of a deficiency of hoſpital mates, when I was lately ſent to act for a little while at the general hoſpital at the Helder, when the wounded of the army were brought, I had an opportunity of witneſſing the Ruſſian management of the ſick and wounded. Here ſhattered ſtumps, compound fractures, deadly wounds, were all promiſcuouſlly left to unaſſiſted Nature; or rather, Nature's intentions appeared to be groſſly perverted by the effect of the ſurgeon's art. The charge of a Ruſſian battalion, of more than a thouſand men, is intruſted to a ſurgeon-major and about half a dozen of mates or aſſiſtants, who are called Feldſhers. Of the former I know nothing, but ſuppoſe they may enjoy advantages both of education and experience; the latter, like the German feldſhers, are a ſet of vulgar lads, mean, and, God knows, humble, who pretend not to the knowledge of diſeaſe; who are only initiated in the art by the dreſſing of ſores, and the adminiſtration of clyſters; and belong to that claſs of human beings formerly known by the name of barber-ſurgeons.

In the Ruſſian hoſpital at Helder, were crowded together in one ſmall apartment about an hundred men, ill of fever and fluxes. In their perſons, the Ruſſians are abominably dirty, which, along with their thick blankets and confined air, (for the windows were cloſely ſhut) preſented a ſcene truly characteriſtic of human miſery and human prejudices. In the apartment of the wounded, from which alſo the external air was as ſcrupulouſly denied as if it had been a poiſon, were about two hundred; they were unuſually crowded; but this aroſe from temporary preſſing circumſtances, and which, by the attention of ſome of the Britiſh hoſpital ſtaff, was, in a few days, remedied, as far as their humanity could go. The Ruſſian ſurgeons were ordered to be ſhewn, and to practiſe, the Engliſh method of dreſſing the wounded, which taſk was far from being deſirable to the teacher; for the ſurgeon, though dignified with the title of major, appeared to hold among the ranks of man but an inferior ſtation. His appearance was truly characteriſtic, and muſt be deſcribed:—A lean, tall figure, dreſſed in a green great coat down to the ground, a leather millitary cap arched with a row of green feathers, having a long white-topp'd cane in its hand, was introduced to us as the ſuperior of the Eſculapian brotherhood. A few broken accents in German, in a weakly, ſhrill voice, told us, he was happy to ſee us, and would be glad of any of our aſſiſtance. On obſerving how neceſſary it would be to preſerve cleanlineſs, and admit freſh air into the hoſpital, he called to ſome orderlies, as if he wiſhed to ſhew he maintained authority; but one of his own officers being preſent, a rough, unfeeling ſon of Mars, in reply to the poor ſurgeon's neceſſary ſuggeſtion, ſoon convinced me how little ſway or re-

ſpect

ſpect any effort of his could have in the œconomy of *his* hoſpital. He appeared afraid of touching a wounded limb, and yet would remove a fractured one from a bed, and put a bandage round it on a ſeat; but if ingenuity had been put on the ſtretch, a dreſſing more unkindly could not be thought of, than that uſed by the Ruſſian ſurgeons. The lint they uſe is the threads drawn out of coarſe Ruſſian cloth, without any preparation; it is hard, and reſembles a ſkein of thread; with this the wounds were plugged up, above which, his digeſtive, in which there appeared to be a great proportion of reſin, was applied; others dreſſed their wounds with vinegar and ſugar. To a perſon of this deſcription it was by no means pleaſant to order a new plan to be adopted, and his own to be laid aſide. The poor man, as well as his aſſiſtants, appeared ſenſible of their imperfections. The contracted allowances granted them by their government, for themſelves and ſick, ſeemed alſo to have contracted their own ſouls.

In the Ruſſian Hoſpital at Helder, were a great number of bad gun-ſhot caſes, from which much information might be drawn; but, from the ſhort time I remained there, cannot *now* make any remarks upon them.

The general want of immediate hæmorrhagy in gun-ſhot wounds is a circumſtance obſerved by all practitioners. It is probable, immediate hæmorrhagy never happens, unleſs when the force of the ball is nearly ſpent, or when a veſſel is partially divided tranſverſely; and this laſt, I ſhould ſuppoſe, can only happen where the ball comes out of the piece truncated, or with edges, as from rifled pieces, when the wound will, in ſome meaſure, partake of the nature of an inciſed wound. Whether this want of hæmorrhagy is always owing to a ſudden and total deſtruction of all life in the injured parts, by which the expoſed arteries refuſe any longer to propel their contents; or, whether it be from the ſudden extraordinary preceding ſtretching, that an extraordinary temporary contraction, with life, follows, appear to me queſtions not to be readily decided.

A limb ſhall be carried off by a cannon-ball; the arteries ſhall be ſeen hanging out, as if they had either been elongated from above, or torn up for inches below, and ſtill with little or no hæmorrhagy. Whether that which follows from ſuch veſſels afterwards be of the active or paſſive kind, I have not yet had ſufficient opportunities of examining.

Is the bleeding which ſucceeds the application of a leech to be attributed to a vacuum formed by its mouth for a conſiderable time over the divided extreme veſſels, deſtroying their tone, ſo that they loſe the power of contracting? or, is this evident loſs of tone rather to be referred to ſomething deleterious in the probofcis proboſcis of the animal, by which it not only renders paſſive the extremities of the divided veſſels, but alſo prevents coagula from forming? There is one circumſtance attending gun-ſhot wounds, which I apprehend may be worthy of notice: The great velocity with which cannon and muſquet balls paſs through the air, muſt occaſion in their rear a momentary vacuum; may not, therefore, much miſchief be done by a portion of our body being expoſed to a ſituation where external preſſure is ſuddenly removed? In thoſe caſes which terminate fatally by the wind, as it is called, of a cannon-ball, that is, where the ball paſſes without touching, it is not likely death is brought about by a ſudden concuſſion of air on a part, (as the expreſſion implies) but by a ſudden removal of a portion of air from a part eſſential to life, by which all external ſupport is removed, rupture of veſſels, or other miſchiefs follow, terminating in death; and this event à priori will be moſt likely to happen when the ball paſſes acroſs the head, cheſt, or epigaſtric region. There is another fact tending to the ſupport of this idea, which is, that a touch with a cannon, or even a muſquet-ſhot, though hardly ſufficient to diſcolour the ſkin, will yet ſometimes knock the perſon down. It has alſo been alleged, and, poſſibly, not without reaſon, that ſlight touches from cannon or muſquet ſhot, are attended with more tedious or dangerous conſequences than apparently ſimilar touches from another mode of infliction; if this be really the caſe, it may probably ariſe from a vacuum ſuddenly formed over veſſels, thereby injuring or deſtroying their tone. Laſtly, a blue line, or mark, is ſometimes obſerved on men after actions, although they were not ſenſible of a ball having touched them; the extravaſation here, then, was probably owing to the external ſupport of the air being taken off for an inſtant, occaſioning rupture of veſſels, and the effuſion mentioned. But it has been alleged, and ſometimes, perhaps, alſo, with reaſon, that people engaged in action, where the grim meſſengers fly thick, may be occaſionally ſo overpowered with horror that a very ſlight blow will be ſufficient to make them tumble; or, as others will have it, and which I ſuppoſe to be moſt likely, as well as moſt frequent, the imagination on ſuch occaſions may be ſo much heated by momentary impulſe and ardour poſſeſſing the whole ſoul, as to deprive men of their acute feelings, and thus a ball may graze them without their knowledge.

[ To be continued in our next Number. ]

